# Developing scale for affective response: anxiety, anger, depression

**DOI:** 10.1192/j.eurpsy.2024.1093

**Published:** 2024-08-27

**Authors:** H. Y. Shin, D. Y. Kim, S. W. Choi

**Affiliations:** Clinical Psychology, Duksung Women’s University, Seoul, Korea, Republic Of

## Abstract

**Introduction:**

People experience various negative emotions when they encounter stressful events, and these negative emotions contribute to the onset of illnesses. These emotional responses are not limited to just one; a person can experience multiple emotions at once, and the primary emotional reactions can vary depending on the severity and duration of the illness or life events. This is reason why we created a self-report scale to assess short-term emotional responses, focusing on the current emotional state experienced subjectively by patients.

**Objectives:**

The purpose of this study was to develop an affective response scale (ARS) and examine its validity and reliability.

**Methods:**

We established clusters of affective via a literature review and developed preliminary items based on the structure. We conducted expert content validation to converge on the final items, followed by construct validity and reliability analyses.

**Results:**

The research findings indicate that the Affective Response Scale was composed of three main dimensions: anxiety, anger, and depression. Content validity results confirmed the validity of most items. The scale developed in this study was found to be valid in both exploratory and confirmatory factor analyses, and it was identified to be stable and consistent through the analysis of the internal reliability.

**Conclusions:**

These results indicate that the ARS is highly reliable and valid, and that it can be utilized as an effective measure of the patient’s emotion and its severity.

**Disclosure of Interest:**

None Declared

